# Quantifying biologically essential aspects of environmental light

**DOI:** 10.1098/rsif.2021.0184

**Published:** 2021-04-28

**Authors:** Dan-E. Nilsson, Jochen Smolka

**Affiliations:** Lund Vision Group, Department of Biology, Lund University, Sölvegatan 35, 22362 Lund, Sweden

**Keywords:** radiometry, environmental light, environmental light field, measurement method, camera, human vision

## Abstract

Quantifying and comparing light environments are crucial for interior lighting, architecture and visual ergonomics. Yet, current methods only catch a small subset of the parameters that constitute a light environment, and rarely account for the light that reaches the eye. Here, we describe a new method, the environmental light field (ELF) method, which quantifies all essential features that characterize a light environment, including important aspects that have previously been overlooked. The ELF method uses a calibrated digital image sensor with wide-angle optics to record the radiances that would reach the eyes of people in the environment. As a function of elevation angle, it quantifies the absolute photon flux, its spectral composition in red–green–blue resolution as well as its variation (contrast-span). Together these values provide a complete description of the factors that characterize a light environment. The ELF method thus offers a powerful and convenient tool for the assessment and comparison of light environments. We also present a graphic standard for easy comparison of light environments, and show that different natural and artificial environments have characteristic distributions of light.

## 1. Introduction

In diverse fields such as architecture, lighting science, visual ergonomics, environmental psychology and visual ecology, measurements of environmental light are often essential. The methods used to measure light in these fields are based on long traditions and often selected to be as convenient as possible. We perceive the light environment around us solely through our eyes. From this, it would seem obvious that methods for quantifying light environments sample similar types of information as the eye does. But the current methods for measuring environmental light are typically rather indirect for assessing the light reaching our retina [1–3]. A striking example is the extremely widespread use of lux-meters. These measure illuminance with an upward-directed cosine-corrected angular sensitivity [1]. Yet, there is nothing in the eye that even remotely acts like a cosine-corrected detector. Illuminance readings quantify the light that reaches the environment, usually from above, whereas our experience of a light environment is based on light reaching the eye after it has been reflected, refracted or transmitted by the environment. This is a fundamentally important distinction, because the visual impression we get from the environment is not primarily concerned with the light sources, but with the objects, materials and surfaces that are illuminated.

In addition, illuminance measurements assume that environmental light can be represented by a single value as if reading the temperature of the environment. However, the fact that we see structures and objects around us is because even in a single visual scene, the radiance varies by two orders of magnitude from the dimmest to the brightest directions, or over five orders of magnitude if light sources such as lamps or the sun are included [4–7]. Environmental light thus varies enormously depending on the direction it comes from. This means that any assessment of a light environment should be made by radiance readings in different directions from typical observation points in the environment. Digital cameras are ideal for conveniently collecting radiance readings from many directions in the environment.

Another problem with current methods for measuring environmental light comes from the standardized (CIE, photometric) spectral sensitivity, the *V*(*λ*) function [8] on which units such as lux and candela are based. In lighting, this spectral sensitivity is generally taken as representing human vision, although it is in fact only the spectral sensitivity of the retinal channel for achromatic contrast, measured in the retinal centre [8]. It completely ignores the blue cones and the retinal channels for colour vision [9–11], i.e. it only targets one of several subsystems of human vision and severely underestimates the spectral width of human vision. Photometric units also incorrectly assume that eyes measure the energy content of light, whereas all biological photoreceptors measure photon flux. This is not an insignificant problem because over the complete visible spectrum from 400 to 700 nm, photon energy differs by 75%. There is also a mismatch between the eye and current methods employed for spectral assessment of the light environment. These methods almost invariably provide high spectral resolution but low (if any) spatial resolution [3,12–16], whereas the eye samples the visual world in quite the opposite way.

Obviously, quantifications of the light environment should consider the properties of photoreceptors in the retina. The human eye contains three types of cone photoreceptors peaking at 437, 533 and 564 nm, which we use for colour vision [9,10,17–19]. We also have rods peaking at 498 nm [9,10,17], which we use for dim light vision. All human photoreceptors have broad spectral half-widths of roughly 100 nm [20]. Apart from rods and cones, the eye contains intrinsically light-sensitive retinal ganglion cells (ipRGCs) peaking at 480 nm [2,21–23]. These have received much attention because of their involvement in non-visual control of the sleep hormone melatonin [2]. The function of the ipRGCs is to acquire information about absolute radiance levels. There are at least five different subtypes of ipRGCs [24], and not all are known to be involved in melatonin control. Rods and cones, on the other hand, measure only relative radiances. Here, information about the absolute radiance is largely removed by several mechanisms (photopigment bleaching, dynamic gain control of the transduction cascade and centre-surround neural integration [9,10,25–27]).

Even though the properties of rod/cone and ipRGC pathways are very different, their strict assignment to visual and non-visual roles now seems to be an oversimplification. The visual pathways involving the thalamus and the visual cortex of the brain also receive input from ipRGCs, and the non-visual pathway, involving the hypothalamic supra-chiasmatic nucleus, also receives spatial information from visual photoreceptors [28–31]. This opens up for the hypothalamic (subconscious) pathway to use image information for basic biological tasks such as selection of a suitable environment (habitat selection), positioning within the habitat and mood setting for general prioritizing of behaviours, i.e. tasks that can be assumed to be directly connected to our mood, wellbeing and to preferences for different environments.

Vertebrate (and human) eyes also harbour other light-sensitive molecules such as neuropsin, peropsin and RGR-opsin, all with unique spectral sensitivities and roles that are gradually being revealed [32–34]. These opsins, together with those of ipRGCs, rods and cones, imply that our eyes detect light differently depending on wavelength across a broad spectral band from 400 to 700 nm. With many of the different opsins being unevenly distributed over the visual field [35–37], and spatial information contributing even to pathways previously thought to be ‘non-visual’ [28–31], it is clear that current methods for measuring environmental light are becoming increasingly obsolete [2].

It is thus urgent to develop biologically relevant ways for quantifying light environments. In this paper, we take our perspective from visual ecology, and assume that the human eye through evolution has been tuned to efficiently pick up biologically relevant information from natural scenes. From this perspective, we identify the light-environment properties that are likely to be of biological importance. Absolute radiances are of course important because they control levels of the sleep hormone, melatonin, through the ipRGCs, but they also set visual performance limits, such as spatial acuity, temporal resolution and contrast sensitivity for both luminance and colour [38].

With the exception for some bias depending on whether the dominating light source is behind or in front of the observer, environment radiances can be expected to be randomly distributed around the azimuth, but because there are more light sources above than below the horizontal plane in both outdoor and indoor environments, the distribution of light can be expected to vary systematically with the elevation angle. This dependence of light intensity on the elevation angle is a sadly neglected but fundamental feature of all light environments. Also, within each elevation angle of a scene, the light intensity varies to form the contrasts that we use for vision. The magnitude of these contrasts is another neglected variable that has a major and obvious impact on the experienced light environment. Also, the spectral balance varies with elevation angle and constitutes an essential aspect characterizing the light environment. Here, it is important to note that the eye samples the spectral content very coarsely (using only three spectral channels for our trichromatic colour vision).

A biologically relevant method for quantifying light environments should allow for simple comparisons of absolute radiances as well as relative radiance differences between and within environments. The method should be applicable to single scenes as well as to entire environments with sampling at multiple vantage points. Here, we describe a method which combines all these requirements and show that it successfully discriminates between different types of natural and artificial light environments, different times of day, seasons and weather conditions.

## 2. Results

**Figure 1.**
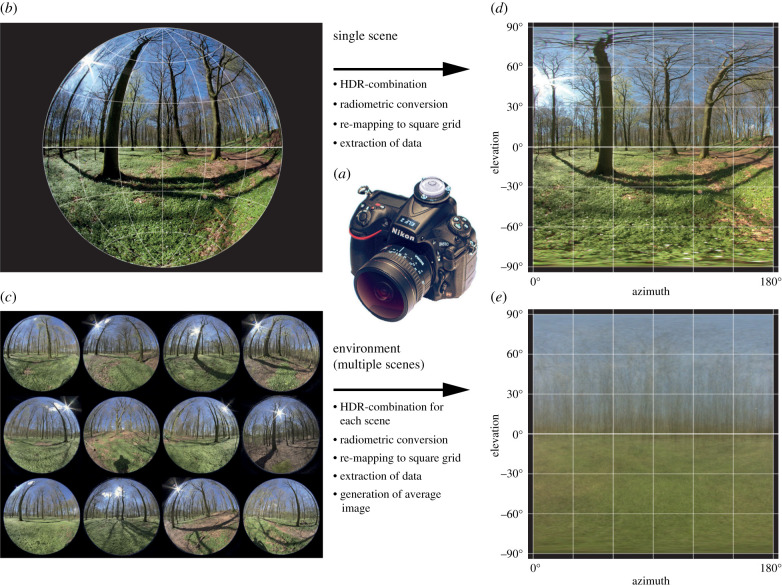
Quantification of environmental light with the environmental light field method. Data are acquired with a digital camera equipped with a 180° fisheye lens (*a*). By means of a mounted bubble level, the camera is kept horizontal during exposures. The hemispherical field of view generates a circular image where different fisheye lenses may have different projections. We used a Sigma 8 mm/F3.5 with an equisolid projection (*b*). To cover the full dynamic range of natural scenes, different exposures are taken of each scene (typically three exposures with ±3 EV bracketing), and the images from each scene are combined into a high-dynamic-range (HDR) image. Measurements can be based on a single scene (*b*) or multiple scenes representing an environment (*c*). The circular projection is remapped into a square projection (*d*) where elevation and azimuth angles are orthogonal. The top of these images represents straight up and the bottom straight down, with the horizontal plane in the middle. For measurements with multiple scenes an average image is generated (*e*).

To quantify the biologically most relevant aspects of light environments we developed a technique based on a digital camera with a 180° fisheye lens. For the recording of light environments, the camera is held horizontally (by means of a simple bubble level) such that the fisheye optics cover all elevation angles from straight up (+90°) to straight down (−90°) with the horizontal plane running through the centre of the image (figure 1). This allows simultaneous recordings of as many radiance values as there are pixels within the circular image of the fisheye lens. Each such image can be said to contain information of the light distribution in a single scene, which may be relevant for visual ergonomics, for assessing changes in the light environment over time or for comparing different types of lighting.

For analysis of a complete environment, such as a room or a forest, where people can move around and experience the environment from different vantage points, we use multiple exposures (typically 10–40) from different positions within the environment (figure 1). This has the advantage of producing general measurements which are representative for arbitrary positions in the environment, and where specific features of individual scenes can be removed by averaging.

The images contain radiance information for different elevation and azimuth angles, but because the hemispherical field of view is imaged as a circle, with a lens-specific projection, we remap the images to an equirectangular format where elevation and azimuth angles are orthogonal (figure 1). For environments measured by multiple scenes, we derive an average remapped image. This will stretch the small solid angles in the upper and lower extremes of the images but has no consequences for the mathematical weighting of different elevation angles.

To cope with the large dynamic range of natural scenes, a single exposure is rarely sufficient, and may result in significant proportions of saturated or completely black pixels where the actual radiance is unknown. Accidental over or under exposure can make this problem severe. The obvious remedy is bracketing with several different exposures of each scene. We found that three exposures, each separated by 3 EV units (a factor 8), are sufficient to cover the dynamic range of radiances within single scenes. Pixels aimed at light sources such as the sun's disc may still be overexposed, but that is true also for the image in our retina. The different exposures are combined into a high-dynamic-range (HDR) image.

Environmental light in general can be expected to be distributed over elevation angles in environment-specific ways, whereas the radiance distribution over azimuth angles is more random. For this reason, we use the remapped images (figure 1) to derive radiance information as a function of elevation angle. We deem the dependence on elevation angle as essential for how we are influenced by light and how the environment is perceived. For any elevation angle, there is of course a range of different radiances at different azimuth angles. Rather than computing the average, we use the median radiance, because it better represents typical radiances, and is less influenced by strong light sources covering very small parts of the environment.

The range of radiances at each elevation angle is an informative feature related to the visual contrasts and with an obvious impact on our perception of the light environment. To quantify the range of radiances at each elevation angle we compute narrow and wide confidence intervals containing 50% and 95% of the radiances. From experience, we have found that a 3° resolution of elevation angles is sufficient to bring out characteristic and important features that differ between environments. With image sensors of at least 1 MPix, this allows for spatial contrasts to be assessed at all orientations.

The spectral channels sampled by typical RGB image sensors have bandwidths similar to the spectral channels of animal and human vision (50–120 nm half-widths) [7], providing RGB cameras with a spectral resolution similar to that of animal vision. We thus compute the radiances (median with contrast-span envelopes containing 50% and 95% of the values) separately for red, green and blue channels (the spectral characteristics of the colour channels are described in the electronic supplementary material and also considered in the Discussion). We also compute radiances for white light integrated over all three spectral slots. The computed radiance parameters are summarized in table 1.

**Table 1.** Parameters calculated for each scene or environment (making a spreadsheet of 60 rows and 37 columns).

for each 3° span of elevation angles
for the wavelength bands white (400–700 nm), red (600–700 nm), green (500–600 nm), blue (400–500 nm)
mean radiance
radiance standard deviation
median radiance
25th percentile
75th percentile
2.5th percentile
97.5th percentile
minimum radiance
maximum radiance

To agree with the way human and animal eyes detect light, we calibrate the camera's exposure and pixel values to photon flux radiance. The unit is based on photon flux per area, solid angle and spectral range: photons s^−1^ m^−2^ sr^−1^ nm^−1^. For daylight or even indoor light this results in big numbers in the range 10^13^–10^18^, and the total range from starlight to sunlight covers nine orders of magnitude. To handle both the big numbers and the huge range of naturally occurring values we use log_10_ values to describe radiances. For convenience, we use the abbreviation ‘lit’ for the intensity unit 1 log quanta s^−1^ m^−2^ sr^−1^ nm^−1^ (to avoid the impractical spelled out unit: log_10_ number of photons per second per square metre per steradian per nanometre wavelength). This results in manageable values of median radiances ranging from 18 lit in bright sunlit scenes, and down to 9 lit in starlit scenes. Even though the spectral component of the unit is per nanometre wavelength, we integrate over the broad spectral bands of the RGB image sensor. This unit implies comparable radiance values for arbitrary spectral bandwidths.

**Figure 2.**
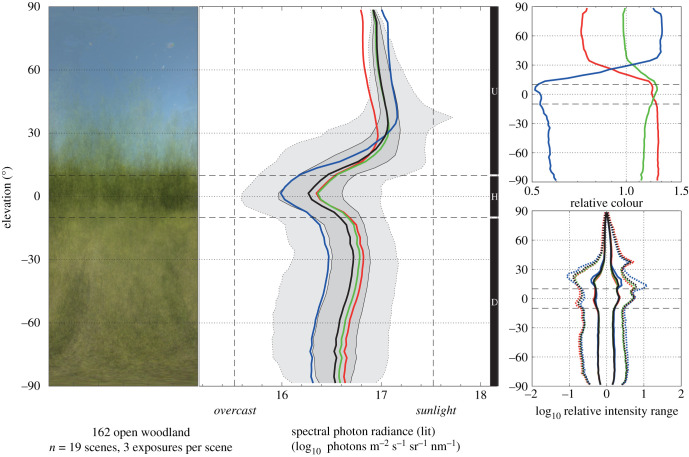
Presentation of ELF data. The orthogonal remapped images from figure 1 are used to generate radiometric data as a function of elevation angle. Pixel values and exposure data are converted into photon radiance in the unit: log_10_ quanta m^−2^ s^−1^ sr^−1^ nm^−1^ (for convenience denoted ‘lit’). For each 3° segment of elevation angles, the median photon radiance is calculated from radiometric calibrations for blue (400–500 nm), green (500–600 nm) and red (600–700 nm) spectral slots, as well as for white light (400–700 nm). We use the median rather than mean values to represent typical radiances and to minimize the influence of strong light sources covering only a small number of pixels. In addition to median radiances, we compute the range of radiances containing 50% and 95% of all pixel values within each 3° slice of elevation angles. The figure shows a standardized presentation of measurements that was developed to allow for easy assessment and comparison of different light environments. To the left, this has a remapped image (laterally compressed) of the scene or an average image in the case of an environment with multiple scenes. In the centre, a main diagram displays radiances as a function of elevation angle. Red, green and blue spectral bands are represented by coloured traces, with a black trace for the full spectral width, and the range of radiances (the contrast-span for white light) for 50% (dark grey) and 95% (light grey) confidence intervals. To the right are two diagrams where the spectral composition (upper) and contrast-span (lower) are highlighted by normalization to the white light curve (black trace in the central diagram). These graphs make it easy to see how the colour distribution and contrast-span vary with elevation angles because the information is not superimposed on the absolute intensity curve. A simplified representation of the same light environment is found in table 1. The environment is a sunlit open woodland with a typically dark horizontal band separating different spectral compositions above and below the horizon.

To facilitate comparison of light environments, we use a standard chart (figure 2) containing four panels: (i) a horizontally compressed version of the remapped image, or average image, (ii) a main diagram with absolute radiances (*x*-axis) as a function of elevation angle (*y*-axis), plotted as median values for RGB spectral bands, and for white light together with its contrast-span, (iii) a small diagram highlighting the spectral composition as a function of elevation angle and (iv) a small diagram highlighting the contrast-span as a function of elevation angle. The colour and contrast-span information are contained also in the central diagram but made clearer in the two small diagrams by normalization to the absolute radiance curve for white light. For applications that do not need the resolution in elevation angle, the software also computes a simplified table with average radiance, contrast-span and RGB ratios in the upper and lower field of view (table 2).

**Table 2.** Simplified presentation of ELF data. Four text lines provide numbers for the white-light median radiance, integrated over the upper field +10° to +90°, and the lower field −10° to −90°, together with the associated intensity range (contrast-span) containing 95% of all values, and the relative percentages of red, green and blue. Bold indicates the most important values.

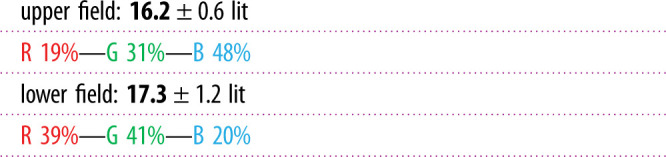

Using the environmental light field (ELF) method with the standard charts (figure 2), we have quantified over 1000 natural light environments across the world, on five continents, from dense forest to deserts, from alpine to maritime environments, at all times of day and night, in different seasons and under different weather conditions. We have also measured rural, suburban and urban environments as well as a broad selection of indoor environments. Most of this database of light environments will be presented and analysed elsewhere. Here, we describe a few samples for the purpose of evaluating and testing the method.

We found measurements with multiple scenes to be superior for characterizing the general properties of an environment. By including 10–40 scenes, specific features unique to single scenes (see electronic supplementary material, figure S10) are effectively averaged out, revealing the typical features of the environment. Including more than 40 scenes does not improve or change the results, but obviously increases the risk that the light environment changes during the time it takes to sample all the scenes. Single scene measurements are useful mainly for analysing changes in the light environment over time, between different weather conditions or with different lighting, and also for assessing visual ergonomics of a workplace or similar.

**Figure 3.**
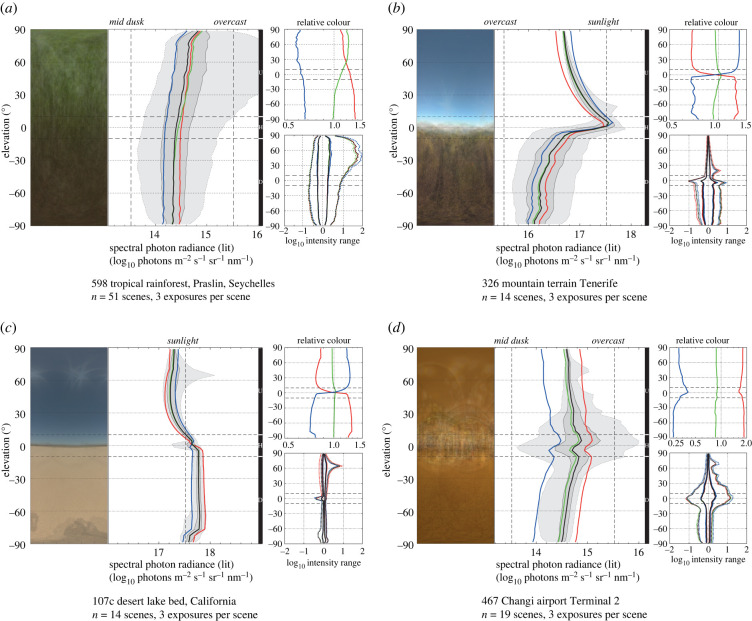
Comparing different environments. ELF charts of light environments measured (*a*) at a dense tropical rain forest at the Seychelles, (*b*) in mountain terrain at Tenerife island, (*c*) on a desert lake-bed in southern California, all close to noon under sunny conditions and (*d*) indoors at Changi airport, Singapore. Together with the open woodland of figure 2, these light environments all have their own unique features revealed by the different components of the ELF chart.

Daytime outdoor environments with different degrees of openness such as the open woodland in figure 2, the mountain terrain in figure 3*b*, and the dry lake-bed in figure 3*c* reveal the typical spectral distinction between sky and ground, where the sky is dominated by blue light and only has low levels of red light, whereas the ground is dominated by a mixture of green and red but has comparatively little blue. In the open environments of figures 2 and 3*b* the contrast-span is obviously low at elevation angles with clear sky but high around the horizon and the lower field. Open woodland (figure 2), savannahs and shrubland typically display a dark band around the horizon, whereas high altitude open terrain (figure 3*b*) may instead feature a distinctly bright band just above the horizon. The sky is generally brighter than the ground, but in the dry lake-bed (figure 3*c*) low air-humidity and a lack of vegetation cause the ground to instead be brighter than the sky. The dense tropical rainforest (figure 3*a*) stands out because practically no sky is visible, there is no obvious horizon and the contrast-span is particularly wide above the horizontal plane. In the indoor plaza environments (figure 3*d*), red dominates at all elevation angles and the contrast-span is wide close to the horizontal plane. Despite the large differences in the above light environments, no single variable can be used to discriminate between them. But with all the ELF variables together, each environment is characterized by its own unique profile.

**Figure 4.**
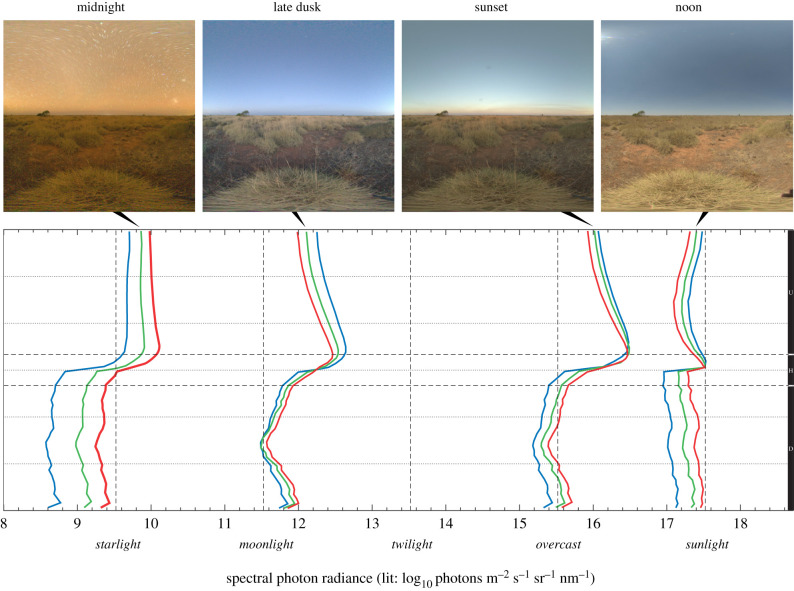
Light environments changing over the day. A single scene recorded over 24 h from a Western Australian desert, during new moon, clear skies and without light pollution at night. On the radiance diagram covering more than 10 orders of magnitude, RGB radiance traces are shown for four different times from the brightest day to the dimmest night, together with the HDR images of the measurements. Apart from the huge differences in absolute radiance, the curves also reveal systematic differences in spectral composition and relative radiance distribution at different elevation angles. The dashed levels of starlight, moonlight, twilight, overcast and sunlight are arbitrary indications intended to aid orientation on the radiance scale. These are used also in the standard ELF diagrams.

**Figure 5.**
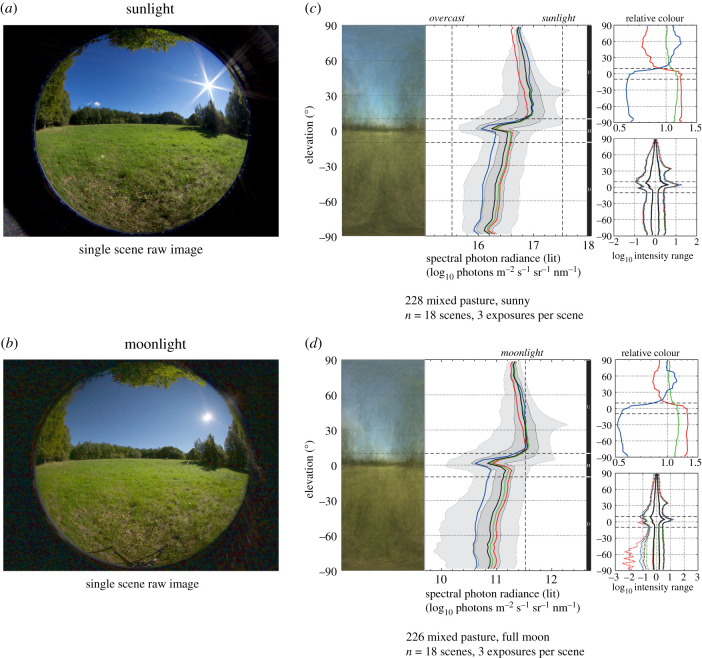
Sunlight versus moonlight. By waiting 12 h at equinox and full moon, the sun and moon will swap positions in the sky. The pasture scene in (*a*) and (*b*) demonstrates how similar moonlight is to sunlight, although the absolute radiance differs by five to six orders of magnitude. Measurements in (*c*) and (*d*) show an environment composed of 18 predetermined scenes, including the one in (*a*) and (*b*). Here, the shapes of the radiance curves are almost identical in sunlight and moonlight, despite the large difference in absolute radiance. Also, the spectral and intensity range (contrast-span) diagrams are similar in sunlight and moonlight, although moonlight is less rich in blue. There is also an artificially increased contrast-span in moonlight caused by dark-noise in the red channel.

The time of day in outdoor environments (figure 4) has an obvious impact on the absolute radiance, but also affects scene contrast, the relative intensity of sky and ground, and the spectral balance. The intensity difference between sunlight at noon and a starlit night exceeds 8 log units, and starlight is also distinctly red, whereas twilight under a clear sky is more blue-dominated than during any other time. The absolute radiance changes significantly over 24 h but the relative radiance of sky and ground remains similar under sunlight and moonlight. The comparison of sunlight and moonlight in figure 5 was made during full moon, 12 h apart at equinox, meaning that the sun and moon occupy the same position in the sky. Since the sun and the moon are nearly the same size and both generate blue skylight by scattering, the two light environments are almost indistinguishable apart of course from the large difference in absolute intensity. Here, the benefit of the logarithmic unit is clearly revealed: the shapes of all the curves are close to identical, describing the relative distribution of light in the environment, but the placement of the curves on the intensity scale reveals the more than 5 log unit difference in absolute intensity.

**Figure 6.**
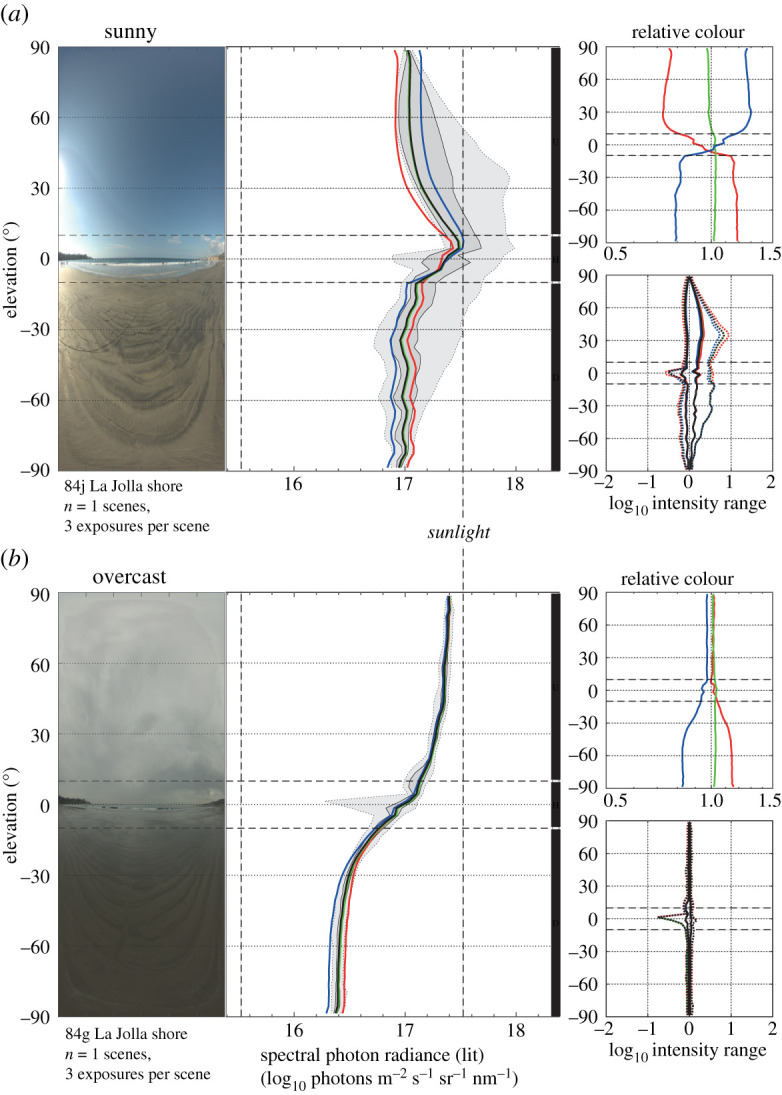
The impact of weather conditions on the light environment. The same scene from La Jolla beach, San Diego, under (*a*) sunny and (*b*) overcast conditions. Apart from a relatively darker ground, overcast conditions cause a massive reduction in contrast-span at all elevation angles.

Weather conditions can dramatically change the light environment (figure 6). A change to overcast conditions implies that the ground becomes much darker than the sky, the blue dominance in the sky is lost and scene contrast is dramatically reduced. We note that environments that appear dull typically have very narrow contrast-spans. We also note that time of day and weather conditions have consistent effects on most outdoor environments. The general conclusion is that the ELF method successfully can quantify and distinguish between different environments as well as between different conditions.

Assessments of measurement errors (see electronic supplementary material) reveal that random exposure variation of the camera and non-ideal spectral shapes of the RGB channels are the main causes of inaccuracy. Total errors are typically below 25% (0.1 lit) but may be twice this under unfavourable conditions. However, these errors are not large enough to compromise the characterization and discrimination of light environments. Both the spectral errors and the random exposure variation are due to technical limitations of the consumer cameras we used, and we assume that consumer cameras of other models or from other manufacturers would generate similar errors. It would be technically straightforward to construct a devoted ELF camera with a measurement accuracy reduced to a few per cent. Spectral channels with ideal (steep) band-pass characteristics could be achieved by a broadband monochrome image sensor behind a spinning disc of band-pass filters of much larger dimension than those allowed by the Bayer mask of RGB image sensors.

## 3. Discussion

It is not immediately obvious which features to include in a method for quantifying environmental light. Here, we have taken a biological approach and aimed at features we know the eye can pick up. First and foremost, this means measurements of radiance (light reaching the measurement device from the environment) and not irradiance (light illuminating the environment). We have aimed to minimize the number of parameters and the resolution, to keep down the amount of data, but without compromising the ability to discriminate between light environments that we perceive as different or that may influence us differently in other ways. We initially tried to include analyses of spatial frequencies and various aspects of higher orders of image statistics, only to find that this information was more confusing than helpful. Instead, we found that measuring radiance as a function of elevation angle is a key principle that also agrees with the way human and animal retinas are structured [35,39–41]. By quantifying absolute radiance as median values, contrast-span and distribution over three spectral channels, all as functions of the elevation angle, it is possible to find quantitative differences between light environments we perceive as different. We have aimed to structure the rather complex radiometric data to suit the widest possible application to visual science, interior lighting, architecture and visual ergonomics. Admittedly, the amount of data may still seem overwhelming, but to fully characterize and compare light environments, all the parameters are needed. The simplified data presentation standard (table 2) is useful in applications where the fine resolution of elevation angle is not needed.

Photometry and radiometry already abound with different units of measure. We here argue that introducing a logarithmic unit of photon flux has important merits. The typical radiance values for outdoor sunlit environments are 16–18 lit, overcast 15–16 lit, indoor lighting 13–16 lit, moonlight 11 lit and starlight 9 lit. Because of the logarithmic unit, radiance values never become very large or very small. The difference between sky and ground is often less than 1 lit. Within a complete scene or environment, the total intensity range is typically around 2 lit (a factor of 100 from the brightest to the dimmest direction, excluding light sources). The total range from the dimmest part of the dimmest environment to the brightest part of the brightest environment is about 10 lit (a factor of 10 billion). Expressed in the unit of lit, this range may seem numerically small, but it has the advantage that three significant digits (two decimal places) offers sufficient accuracy for typical measurements: an increase or decrease of 0.01 lit implies a linear change of only 2.3%, which is hardly perceived, given the human threshold for simultaneous contrast of 1% in bright light [42]. Even just two significant digits would often be sufficient to describe and accurately recreate a light environment because humans are remarkably poor at recalling absolute radiances [43].

The use of median radiance values rather than averages allows for a more representative description of radiance levels. Average values would be strongly influenced by concentrated light sources even if they just cover a small number of pixels. This influence would also depend heavily on the degree to which these pixels were oversaturated. By using median values, the possible saturation of a small areas of the image will have no effect. Median radiances have the benefit of pointing to typical or common pixel values, because there are equal numbers of brighter and darker pixels.

Does the ELF method include all quantifiable aspects of light in an environment? The answer is clearly no. In terms of the power and orientation of spatial frequencies, much more data could be extracted from the recorded images, but we have only included a simple contrast-span parameter because it quantifies the dynamic range facing a visual system. More detailed information on the distribution or orientation of different spatial frequencies would say more about the visual information in a scene than it would about the general light environment. Likewise, higher-order image statistics [44–46] are associated with the visual information content rather than with the light environment under which the visual information appears. The features that should be considered to contribute to a light environment are of course a matter of definition. Here, we argue that the distribution of light over elevation angles, including its coarse spectral composition and its contrast-span (table 2) are the key features that characterize a light environment. These are all obvious and quantifiable aspects of light, and together they are efficient in distinguishing between different light environments. It of course opens up for environmental psychology to test whether the ELF method quantifies aspects of environmental light that are important for our perception and wellbeing. A strength in this respect is that the acquired images can be saved, allowing for later extraction of other types of information that unexpectedly may turn out to be important.

The three spectral channels of RGB image sensors are suitable for assessing environmental light for humans [47], but depending on species, animals have different sets of photoreceptors in their eyes, covering different spectral ranges [26]. It is technically straightforward to use more or narrower spectral slots, or to extend the spectral analysis beyond the human visible spectrum. Many animals, including birds and insects, have a visual sensitivity extending into the UV (300–400 nm), and quantification of their light environment would require ELF with a UV channel. It is also possible to design the spectral sensitivities to closely mimic different photoreceptor classes in the retina. This has been done for bird vision [48] and could easily be adapted for human vision.

For most of the present investigation, we used a consumer camera which has rather typical spectral sensitivities for the RGB channels, but other makes and models have slightly different spectral sensitivities, and if used for ELF measurements, this may of course lead to slightly different results. To base a method on arbitrary cameras that may differ in their spectral characteristics is obviously unsatisfactory, and selecting a specific camera model is equally unsatisfactory. We thus propose an aim for a spectral sensitivity that has more ideal properties: equal width of all spectral channels, no overlap or gap between spectral bands and equal sensitivity across the width of each spectral channel. An RGB system complying with these requirements and covering the total range 400–700 nm would have a blue channel with a flat-topped sensitivity over 400–500 nm, and equally a green channel covering 500–600 nm and a red channel covering 600–700 nm (see electronic supplementary material). Such spectral curves are obtainable with filters larger than those of the Bayer masks of RGB image sensors, and would require a different technique for acquiring spectral channels (such as a spinning disc of spectral filters in front of a monochrome camera [48]). Using spectrometer data, we found that our measurements with a consumer camera in fact come very close to measurements with the ideal spectral characteristics specified above (see electronic supplementary material). The differences (errors) are not large enough to compromise the comparison of measurements made by ideal spectral channels and those of typical consumer cameras. We thus suggest that the ideal spectral channels with top-hat spectral curves covering 400–500 nm, 500–600 nm and 600–700 nm can be used as a target standard for ELF. However, correctly calibrated consumer cameras, different from the one we used, are also likely to produce measurements very similar to a camera with the ideal flat-topped spectral sensitivities.

In conclusion, the ELF method measures environmental light the way our eyes see it, with a spectral resolution similar to vision, and reveals an understudied but essential dependence on the elevation angles by which light reaches the observer (table 3). It is the first method that quantifies entire light environments. It reveals the features that make light environments appear different at different places, times of day, seasons and weather conditions. It provides a powerful tool for design and control of artificial lighting, for visual ergonomics and for guiding architect decisions on building design, material colour, placement of luminaires and windows, etc. It can also be used for mimicking natural environments indoors, and to reveal which components of a light environment have consequences for wellbeing and health. In addition, it provides an important tool for visual ecology and for controlling the light environment in behavioural experiments on animals, and in animal facilities for science and agriculture. The ELF method cannot replace measurement of colour temperature or colour rendition index because these concern properties of the illuminating light and only indirectly the properties of light reaching the eye. But ELF measurements can fully replace illuminance measurements or spectral measurements as a way of quantifying a light environment. ELF offers a novel and convenient tool that opens the way for vastly improved assessments of light environments.

**Table 3.** Important features of ELF.

measures light reaching the eye
both individual scenes and entire environments can be quantified
measures radiance as a function of elevation angle
provides spectral resolution similar to human and animal vision
quantifies scene contrasts
logarithmic values allow for easy comparison of both absolute and relative light distribution
photon-flux-based unit agrees with human and animal vision
successfully discriminates between environments and light conditions

## 4. Methods

### 4.1. Camera hardware and optics

The measurements require a digital camera with fisheye optics covering at least 180° in all directions (i.e. producing an uncropped circular image). This is similar to solutions for measuring sky radiance [49,50]. For convenience, we have used a consumer camera (Nikon D810 fitted with a Sigma 8 mm f/3.5 EX DG circular fisheye lens). Any digital camera with an RGB image sensor could be used as long as it can record non-compressed raw files and can be fitted with a circular image 180° fisheye lens. The lens must be of fisheye type producing a circular image covering greater than or equal to 180°. Coverage beyond 180° is acceptable, but superfluous. There are a number of different fisheye lenses on the market, and the choice will of course depend on the size of the image sensor, and mounting compatibility with the digital camera.

### 4.2. Calibration

Calibration of the camera (Nikon D810) was carried out with light from an integrating sphere (ISV410-UV, Electro Optical Industries, USA) which combines the light from four xenon-arc lamps to provide a homogeneous bright light output across its 101.6 mm diameter output aperture. The output of the sphere was measured with three factory-calibrated laboratory spectroradiometers: a RAMSES (TriOS Mess- und Datentechnik GmbH, Germany), a GS-1290 (Gamma Scientific, San Diego, USA) and a PR-680 L (Photoresearch, JADAK, New York, USA). Calibration was carried out with all exposure times available on the camera and across the full range of ISO settings. Calibration included rotation of the camera at 10° intervals from 0° to 90° to determine the vignetting of the fisheye lens at three different aperture settings, F3.5, F8 and F22. The RGB pixel sensitivities were calibrated for 100 nm spectral slots: 400–500 nm (blue), 500–600 nm (green) and 600–700 nm (red).

### 4.3. Sensitivity and dynamic range

An important aspect of the method is that it measures the total range of radiances at each elevation angle within a scene. To ensure a sufficiently large dynamic range, we use bracketing with three consecutive images, each exposed 3 full EV steps more than the previous (thus extending the dynamic range by a factor of 64). This safely brings all pixels into the dynamic range, with the exception of the few saturated pixels that directly image brilliant light sources such as the sun or LED chips. It would be possible to extend the bracketing to bring also these light sources into the dynamic range, but for normal assessment of light environments, the saturation of concentrated light sources is not a problem because it mimics saturation of the human eye. In environments with only extended light sources, most image sensors have an intrinsic dynamic range that normally covers all occurring intensities in the scene, but we still recommend bracketing to safeguard against over or under exposure which might cause saturated or noisy pixels that would compromise the measurement. A HDR image is generated by assigning to each pixel the value that it holds in the ‘brightest’ (highest EV) image where the pixel is not saturated.

### 4.4. Measurement procedure

Because the measurement method records radiances as a function of elevation angle it is essential that the camera is kept horizontal during exposures. The equator of the circular images should represent the horizontal plane. Only then will the image cover all elevation angles from straight up (+90°) to straight down (−90°). To facilitate camera orientation, we mounted large bubble levels on the cameras. Exposures would then not be taken by looking through the camera's finder, but simply by confirming that the bubble level indicates the horizontal orientation of the camera.

To measure environments with multiple scenes, suitable vantage points and directions have to be selected. This can be done as randomly as possible, or with deliberate bias for assumed or actual eye positions of people in the environment. To answer specific questions, vantage points can also be selected to predetermined compass directions or to specific direction in relation to dominant light sources. The height above ground at which measurements are taken can be varied between 0.5 and 2 m with negligible effect on the measurements. If the task is to measure the light environment in a forest canopy or from vantage points of small ground-living animals, the camera position will obviously have to be selected accordingly.

### 4.5. Data processing

Once recorded, the images are computationally analysed to extract the radiance values as a function of elevation angle, their variation and spectral composition. As a first step, images are converted to Adobe's DNG format, allowing us to convert raw formats from different camera manufacturers into a single easy-to-process format. The analysis then starts with extracting the linear image information from the DNG file (including cropping the sensor image to active pixels, demosaicing and, for some cameras, the application of a linearization function; see electronic supplementary material), and converting pixel values and exposure data into radiance values according to the calibration files. The only exception is the calibration for different sensitivities in the different colour channels, which, for technical reasons, is performed after HDR calculations. This is followed by an angular remapping of the camera's equisolid image projection (figure 1) to an equirectangular projection by nearest-neighbour interpolation, producing a new array of pixels with equal angular span in the vertical (elevation) and horizontal (azimuth). The resulting images are square, with all pixels from the same elevation angle on straight horizontal lines. Individual bracketing exposures are then combined into HDR images (figure 1) in two steps. First, random inter-exposure differences (which might be due to small errors in aperture opening, or to real differences in light levels between exposures) are eliminated by calculating the median radiance value for each exposure in the bracketing set, and scaling each image to match its radiance to the average across exposures. Second, each pixel in the output HDR image is assigned the value that it holds in the ‘brightest’ (highest EV) image of the bracketing set, ignoring any saturated pixel values.

Single scene measurements are contained in single remapped HDR images, whereas multiple scene measurements are composed of as many images as there were scenes taken in the environment. From this image, or set of images, a number of statistical parameters are calculated for 3° intervals of elevation angle. These values are the median radiance for R, G and B pixels, and radiance ranges (contrast-spans) containing 50% and 95% of the radiance values on either side of the median. The latter values are computed both for the individual colour channels and for their RGB mean. To create an average description of a whole environment with multiple scenes, we calculate the log-average ($\bar{x}=10^{1/n\sum_{i=1}^{n}\log_{10}x_{i}}$, i.e. the geometric mean) of these medians and percentiles across all scenes. In this way, because brightness perception operates on a logarithmic scale, the average of medians reflects more closely a perceptual average. Also, and maybe more importantly, scenes which are less brightly exposed, for example, because a cloud might have moved in front of the sun, still contribute equally to the calculation of intensity variation and range.

All computed values for a measurement, in both single scenes and multiple scene environments, are stored in an xlsx file together with uncompressed TIFF and small jpg versions of the remapped image of single scene measurements and average image of multiple scene measurements (see electronic supplementary material, tables S1 and S8).

### 4.6. Data presentation standard

To allow for easy assessment and comparison of different light environments we developed a standard for graphical presentation of ELF data (figure 2). This contains most of the values computed and stored in the xlsx spreadsheet file (with mean radiances omitted in favour of median radiances). The graphical standard is composed of four panels, including a compressed version of the remapped colour image of the single scene or mean image of multiple scene environments, a main plot of the total radiance as a function of elevation angle, including RGB components and variance (contrast-span envelopes), and two auxiliary plots normalized such as to specifically display the RGB spectral balance and the contrast-span as functions of the elevation angle. Although the auxiliary plots duplicate information from the main plot, they isolate important aspects and are motivated for reasons of clarity. The graphical presentation is in landscape format with print size suitable for fitting two light environments on an A4 or letter page. The graphics for each measurement also contain a name or identification of the light environment together with meta data such as time of day and number of scenes. The Matlab routines provided as a weblink in the electronic supplementary material generate both an editable pdf and a small jpg version of the standard graphics display.

Even with the streamlined graphics display, ELF data may appear complex and overwhelming, reflecting the fact that light environments are composed of many features. To further facilitate presentation and understanding of ELF data, the software also generates a simplified data-table, suitable mainly for comparing and adjusting indoor lighting (table 2). This emphasizes the most important features that in our experience characterize different light environments and allows for basic quantitative comparisons. Instead of plotting the radiance as a function of elevation angle, we here give average values for the upper and the lower fields. The values given are the average radiance for each field for the complete spectral range, 400–700 nm, the 95% contrast-span and the relative RGB contributions in %. The upper field covers +10° to +90° and the lower field –10° to –90°, whereas the horizontal band (±10°), which is less informative, is ignored.

## Software code

An updated version of the Matlab code is freely available at: https://github.com/sciencedjinn/elf.

## Data accessibility

This article has no additional data.
